# Child Marriage and Adolescent Motherhood: A Nationwide Vulnerability for Women in Bangladesh

**DOI:** 10.3390/ijerph18084030

**Published:** 2021-04-12

**Authors:** Jahar Bhowmik, Raaj Kishore Biswas, Sorif Hossain

**Affiliations:** 1 Department of Health Science and Biostatistics, Swinburne University of Technology, Hawthorn, VIC 3122, Australia; jbhowmik@swin.edu.au; 2 Transport and Road Safety (TARS) Research Centre, School of Aviation, University of New South Wales, Sydney, NSW 2052, Australia; 3 Institute of Statistical Research and Training, University of Dhaka, Dhaka 1000, Bangladesh; shossain9@isrt.ac.bd

**Keywords:** child marriage, adolescent motherhood, childbearing, vulnerability, education, religion, consequences, logistic regression

## Abstract

The persistently high prevalence of girl-child marriage and adolescent motherhood is a public health concern in Bangladesh. This study investigated the division-wise prevalence and the influence of education and religious affiliation on child marriage and adolescent motherhood among women in Bangladesh along with their consequences using 15,474 women aged 15–49 years from the Bangladesh Demographic and Health Survey 2017–18. Staggeringly, 82.5% women were married before 18, 43.1% were married before 15, and 61.8% gave birth before 18 years of age. Binary logistic regression models for the complex survey showed that girl-children with primary, secondary, and higher secondary or above education were 16% (95% CI: 0.69, 1.03), 32% (95% CI: 0.55, 0.84), and 87% (95% CI: 0.10, 0.17) less likely to get married <18 years of age, respectively, compared to the uneducated. Also, girl-children with secondary and higher education were 21 and 83% less likely to become adolescent mothers, respectively, than the uneducated. Women married during childhood (<18 years) and adolescent mothers were 36 and 55% less likely to continue studies after marriage, respectively, and expressed that they significantly preferred a late marriage. Policy interventions need to address culturally-laden social norms influenced by religious-related beliefs, especially in rural areas.

## 1. Introduction

Child marriage is a critical public health challenge prevalent across borders and cultures. Child marriage is defined as a customary, formal, or informal marriage or social bond between two person who are under aged, generally 18 years in South Asia, but can vary across jurisdictions [1]. According to UNICEF, 2020, an estimated 650 million women alive today were married as children, and yearly around 12 million girl children were married [2]. Despite a drop of around 11% in child marriage rates in the last ten years in South-East Asia, as of 2020, there were around 30% women married as a child [3], and nearly one in five girls (17%) were married before 15 years of age, making this region particularly vulnerable [1].

Child marriage and adolescent motherhood are intrinsically linked, 90% of adolescent motherhood in the low-and-middle income countries (LMICs) occurred among girls who were already married or in a union [4]. There is a large health and financial burden of child marriage and early maternal age at first birth. Girl-child marriage interrupts the development and wellbeing of women by disrupting or often halting education and future employment opportunities, interpersonal violence, and increased risk of early pregnancy [5,6,7]. Girl-child marriage was found to be a potential reason for early pregnancy, low birth weight of the child, higher child morbidity and mortality [8,9,10]. A report in the USA revealed that women with early first birth experienced less interval between subsequent births, were poorly educated and had fewer assets, lower-income in long-overdue time, and faced greater maternal complications [11]. They are also more probable to risk of anemia, toxemia, and increased risk of neonatal and infant mortality [12]. For example, in South Asia, women aged between 15 and 19 are twice as likely to die due to pregnancy and childbirth related complications compared to women aged 20–24 years [13].

According to the World Health Organization (WHO), 13–17 million mothers gave their first birth at ages under 20 years as of 2020, which was 11% of total worldwide births [14]. The adolescent (15–19 years) first birth rate in LMICs is twice compared to high-income countries, and about 95% of worldwide adolescent births occur in LMICs. More than 50% of world adolescent first childbearing (15–19 years) were in Bangladesh, Brazil, the Democratic Republic of the Congo, Ethiopia, India, Nigeria, and the United States [15]. Bangladesh has one of the highest adolescent (15–19 years) fertility rates, comprising 82 births per 100 women as of 2018 [16]. Thus, a greater characterization of girl-child marriage and adolescent motherhood, especially in LMICs are required for devising potential interventions to improve the quality of life for this vulnerable group of women.

Eight of the 17 Sustainable Development Goals (SDG) set by the United Nations (UN) in 2015 to be achieved by 2030 cannot be attainable without eradicating girl-child marriage [17]. According to a UNICEF report, Bangladesh has the fourth highest rate of child marriage before age 18 among women currently 20 to 24 years old after Niger, the Central Africa Republic, and Chad [18]. The child marriage rate in Bangladesh gradually declined from 66% in 2014 to 59% in 2017 [1,18]. However, the continued high prevalence of child marriage and adolescent motherhood rates could delay the possibility of achieving the SDGs on child and women’s health [7,19,20]. There is a pressing need to evaluate the existing policy for necessary adjustment and targeting intervention programs to keep Bangladesh on track to achieve pertinent SDGs by 2030 before it is too late.

In Bangladesh, a marriage is considered child marriage if the bride or groom is aged less than 18 years [21] and the new Child Marriage Restraint Bill 2017 marriage sets 18 years as the legal age of marriage, but with some leeway for under 18 marriage with parental consent [22]. Early age at first birth or adolescence motherhood defined different thresholds across literature; for example, [23] considered 19 years as a threshold and [24] considered 18 years. The median age of marriage in Bangladesh for those who live in the poorest and richest household were 15, and 18 years, respectively; and 75% of women with no education were married before the age of 18 [25].

The socioeconomic status of women and their household were found to be associated with child marriage in Bangladesh [6,26,27,28]. For example, child marriage was more prevalent in rural areas compared to urban [29]. Another study found that Rangpur and Khulna divisions had more than four times higher odds of being early married as compared to Sylhet [30]. Similar findings were found for adolescent motherhood. Teenage girls from the poorest wealth quintile were more likely to be at risk of adolescent motherhood as compared to teenage girls from the richest households, and the prevalence of early motherhood was more than two times higher among uneducated women than with higher educated women [23]. Ali et al. (2020) [31] observed that early childbearing was more frequent among Muslim women than other groups. While several studies investigated the factors associated with women’s age at first marriage and age at first birth in Bangladesh; there is a literature gap in critically evaluating their subsequent aftermath. Particularly, how being married early or having a child before coming of age impacted continuation of education and how these shaped their outlook on timing their marriage, that is whether women preferred to marry early or later, would need assessment to understand the breadth of social consequences linked with girl-child marriage and adolescent motherhood.

The objective of this study was to conduct a division-wise spatial representation of the estimates of age at first marriage and age at first birth using the latest available Demographic and Health Survey (DHS) of 2017–18 through Geographic Information System (GIS) maps, and to assess their association with socio-demographic factors; thus, the most vulnerable group of women would be identified. Furthermore, the consequences of child marriage and adolescent childbearing on women’s education was to be assessed as well as how these impact women’s opinion on timing their marriage.

## 2. Materials and Methods

### 2.1. Data Description

The Bangladesh Demographic and Health Survey (BDHS) 2017–18 used a two-stratified sampling method to collect nationally representative data for Bangladesh. The sampling frame of the study was set up based on the 2011 Population and Housing Census [32]. In the first stage, enumeration areas (EAs), the primary sampling unit, were extracted from the census information. EAs are based on the lowest administrative areas of Bangladesh: Mahallas (urban) or Mouzas (rural), comprising 120 households on average. A total of 675 EAs (250 from urban and 425 from rural) were sampled using probability proportional to EA size. In the second stage, a systematic sampling of 30 households on average per EA was selected. These resulted in 19,457 households and multiple questionnaires were used for data collection. This study used the women questionnaire that was used to interview 20,127 women with a response rate of 98.8% [32]. The sampled women were aged between 15 and 49 years.

After extracting the relevant variables and cleaning the data, including omission of responses for missing the values (case wise deletion), the final sample size for age of first marriage and age of first birth was 15,674. Due to more missing for post-marriage study continuation and preferred time of marriage, the sample size for this section of the study was 5937, details are given in the next subsections.

### 2.2. Variables

The primary outcome variables for this study were age of first marriage and age of first birth. Both variables were continuous and were categorized to binary variables. For both variables, cut of age was considered 18 years. In Bangladesh, the legal age of marriage for women is 18, although in the new Child Marriage Restraint Bill 2017, marriages below 18 are allowed [22]. This study considered age marriage under the age of 18 as child marriage. For age at first birth, suggestions from [24] were followed, where 18 years were considered “optimal age of first birth” as per evolutionary theories. Thus, two groups were classified: mothers whose first birth was before 18 and otherwise.

The secondary outcomes variables were hypothesized as consequences of child marriage and early motherhood: whether the respondent continued to study after marriage and whether the respondent considered the timing of marriage was early, right time, or late compared to the time respondent married.

Twelve sociodemographic factors and two women’s rights variables were considered as possible predictors. For assessing spatial heterogeneity, district-wise data on outcome variables were also retained. The sociodemographic factors included respondent’s age (≤25, >25); education (none, primary, secondary, higher); wealth index (poorest, poorer, middle, richer, richest); area of residence (rural, urban); geographical division (Dhaka, Barishal, Chattogram, Khulna, Mymensingh, Rajshahi, Rangpur, Sylhet); religion (Islam, others); partner’s age (15–34, 35–50, >50); partner’s education (none, primary, secondary, higher); head of household’s sex (male, female); last 12 month’s working status (not working, working); mobile phone ownership (yes, no); and exposure to media (yes, no). A respondent was considered exposed to media if she read the newspaper, listened to the radio, or watched television at least once a week. The household wealth index, predefined in the BDHS 2017–18, was measured using principal component analysis based on household assets [32].

The two women rights variables considered were whether the respondent considered interpersonal violence (IPV) justified (no, yes) and respondents’ involvement in family decision making (no, yes). The possible causes of IPV that were asked to the respondents were if the wife goes out without telling the husband, neglects the children, refuses to have sex with a husband, or burns the food. If the respondent answered ‘yes’ to any of the causes, then she was classified as someone who considered IPV justified. For family decision-making involvement, four sectors of decision making were asked: health care, large household purchases, visits to family or relatives, and use of money that the husband earns. If the respondent answered that she was involved in any of the decision-making, she was categorized as someone who was involved in family decision-making (‘yes’).

### 2.3. Statistical Analysis

Bivariate distribution of predictors across the outcome variables were quantified as well as primary associations through the Chi-square $\left(\chi^{2}\right)$ tests. The primary binary outcomes were then fitted to the significant covariates obtained from bi-variate analyses using logistic regression models for complex survey designs [33]. For the case of secondary outcomes (study continuation and marriage timing), age of first marriage and age of first birth were taken as covariates, fitted to logistic regression models for complex survey designs as well as adjusted for sociodemographic factors. Preferred timing of marriage was binarized for logistic regression model by collapsing ‘earlier’ and ‘right time’ category and compared with ‘later’ category.

Survey weights, clusters, and strata were adjusted using the *R*-package *survey* (version 4.0). The model provided significance, direction, and effect size of each predictor fitted against the outcome. All statistical analyses were conducted in *R* (version 4.0.3) (Vienna, Austria).

Given the limited literature on child marriage and adolescent motherhood and their consequences in Bangladesh, a *p*-value threshold of 0.005 for discoveries was set as the level of significance criteria in the study [34]. Thus, a predictor was only considered significantly associated if *p*-value < 0.005 and consistent with the relevant confidence interval. Multicollinearity for both models were assessed and generalized variance inflation factor (GVIF) scores were <5 for all variables, which suggested no multicollinearity issue in the models.

Cluster-wise spatial distribution of both primary outcome variables was mapped. The map data was accessed from open-access GIS divisional information. The BDHS provides the location of clusters, an available total of 672, through specific latitude and longitude. However, for the sake of confidentiality, urban clusters were displaced up to 2 km and rural clusters were displaced up to 5 km, with 1% of the rural clusters displaced up to 10 km [35]. The displacement was limited to the second administrative level, that is eight divisional regions in Bangladesh.

## 3. Results

In the BDHS 2017–18, data from 15,674 married women aged between 15–49 years were available for analysis. The women were 32.79 years of age on average with a median age of 32 years. The median age of first marriage was 15.5 years (Mean = 15.9 years, SD = 2.8 years) with minimum and maximum being 9.25 years and 35.9 years, respectively. The lowest age of first birth was 12 years and the highest was 41 years with the median age of first birth was 18 years (Mean = 18.15 years, SD = 3.28 years). Among the respondents, 82.5% of women were married before 18 years of age, 43.1% were married before 15 years of age and 61.8% gave first birth before reaching 18 years of age.

**Figure 1:**
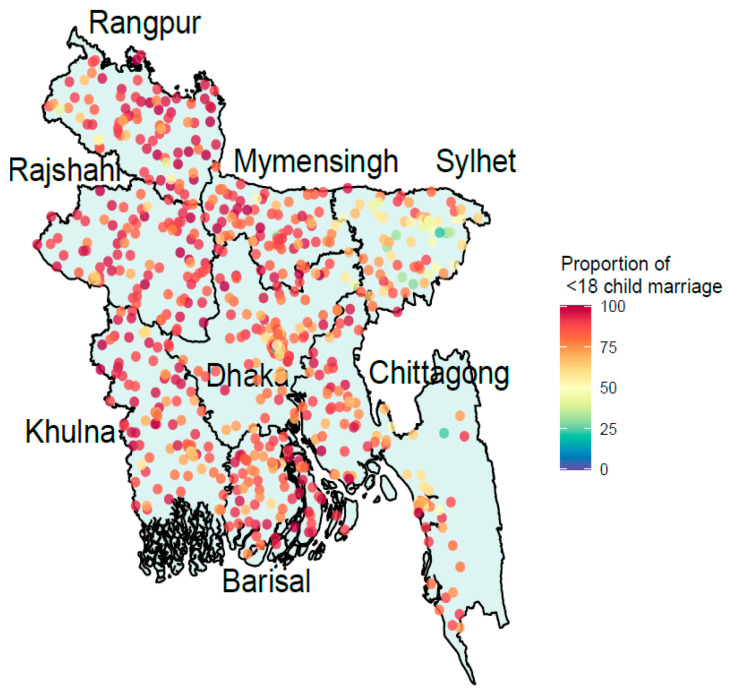
Geospatial distribution of age of first marriage among eight major divisions in Bangladesh.

**Figure 2:**
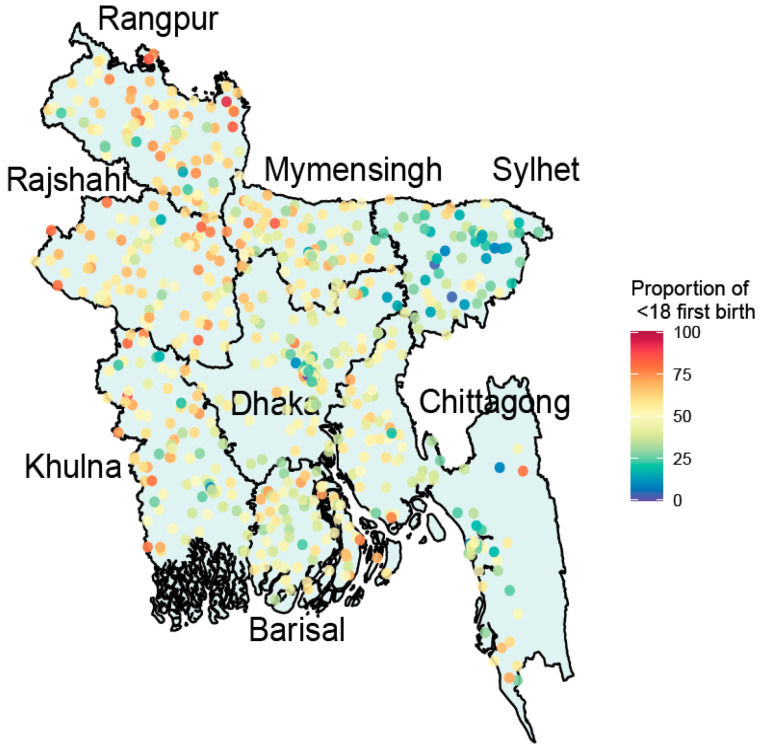
Geospatial distribution of age of first birth among eight major divisions in Bangladesh.

Spatial location using GIS maps presented in Figure 1 and Figure 2 show the distribution of women’s age at first marriage and age at first birth respectively among eight major divisions in Bangladesh. Figure 1 indicates that the percentage of age at first marriage below 18 years of age was highest jointly in Rajshahi and Dhaka (14%), and the lowest was in Sylhet (8.6%). Similarly, Figure 2 shows that percentage of women age at first birth below 18 years of age was highest in Rangpur (14.3%) and lowest in Sylhet (8.2%).

Among the respondents who got married before reaching the age of 18, almost half of them (50.9) were ≤25 years old or younger at the time of the survey, and among the women who experienced first birth during their childhood (<18), 52.7% of them were ≤25 years old during survey time (Table 1). In terms of education, those who got married below 18 years of age, 18.4% of them had no or pre-primary education, 36.1% had primary, 38.9% had secondary, and only 6.5% had higher secondary or above level of education. Of those who experienced adolescent (<18) motherhood, 18.7% of them had no or pre-primary education, 38.1% had primary, 39.2% had secondary, and only 4.1% had higher secondary or above level of education. Regarding the wealth index, 42% of those who had child marriage were from the poor quartiles (poorer and poorest) and 44% of women were from the poor quartiles (poorer and poorest) who had first birth before reaching 18 years of age.

More than two-thirds of women (66.3%) who got married below 18 years old were from rural areas and 67.3% of the women who had experienced adolescent motherhood (<18) resided in rural areas (Table 1). Most of the girl-child marriages (91.6%) and adolescent motherhoods (92.2%) were observed in Muslim households. Table 1 shows that most of the sociodemographic factors such as education, wealth index, area of residence, division, religion, age and education of partner, working status, ownership of mobile phone, exposure to media, and IPV considered were significantly associated with age of first marriage and age at first birth (*p* < 0.005).

Distribution of post-marriage study consequences with age of first marriage and age of first birth are presented in Table 2, and indicates that post-marriage study continuation was significantly associated with age of first marriage and age of first birth (*p* < 0.005). Among the women who were married at <18 years of age, 88.5% of them did not continue to study after marriage and 67.8% of the women who were married above 18 years of age did not continue to study after marriage (Table 2). Similarly, among the respondents, 91.6% of women who gave first birth <18 years of age did not continue to study and 74.2% of women who gave first birth above 18 years of age did not continue to study.

Preferred time of marriage among the respondents was found to be significantly associated with age of first marriage and age of first birth (*p* < 0.005, Table 3); 47.1% of the women who had child marriage and 52% of the women who experienced adolescent motherhood preferred to marry at a later age.

The associations of age of first marriage and age of first birth with potential sociodemographic factors were also assessed using binary logistic regression models after adjusting for survey weights, clusters, and strata-wise variations (Table 4). The association between level of education and age of first marriage was significant for all three levels of education (*p* < 0.005). Women with primary, secondary, and higher secondary or above education were 16% (AOR = 0.84; 95% CI = [0.69, 1.03]), 32% ((AOR = 0.68; 95% CI = [0.55, 0.84]). and 87% (AOR = 0.13; 95% CI = [0.10, 0.17]) less likely to be married at <18 years of age, respectively, compared to their illiterate counterpart (Table 4). Also, age of first birth was associated with the level of education for secondary and higher levels, women with a secondary and higher secondary or above level of education were 21% (AOR = 0.79; 95% CI = [0.69, 0.90]) and 83% (AOR = 0.17; 95% CI = [0.14, 0.20]) less likely to have first birth at <18 years of age (Table 4) compared to illiterate women. Partner’s education also found to be associated with age of first marriage and age of first birth, women with partners having secondary and higher secondary or above levels of education were 24 and 48% less likely to be married at childhood (<18), respectively. Similarly, women with partners having secondary and higher secondary or above levels of education were 15 and 41% less likely to experience adolescent motherhood (<18), respectively.

Area of residence was found to be significantly associated with age of first marriage, women living in urban areas were 18% less likely to be married under 18 years of age (AOR= 0.82; 95% CI = [0.71, 0.94]) compared to women living in rural areas. The religion of women was also found to be significantly associated with age of first marriage and age of first birth (*p* < 0.001). For the women, those who were not Muslim were 63% less likely to be married at <18 years of age (AOR = 0.37; 95% CI = [0.29, 0.46]) and 49% less likely to experience first birth at <18 years of age (AOR = 0.51, 95% CI = [0.42, 0.63]) compared to Muslim women.

Division was significantly associated with age of first marriage for all divisions except for Chattogram, women resided in Rangpur and Khulna division were twice as likely to have child marriage compared to women who resided in Dhaka, and women who resided in Rajshahi, Barisal, and Mymensingh divisions were 89, 57, and 47% more likely to be married at <18 years of age than women that resided in Dhaka division (Table 4). However, women who resided in the Sylhet division were 60% significantly less likely to be married at <18 years of age (AOR = 0.40, 95% CI = [0.30, 0.52]) than women who resided in the Dhaka division. Division was also significantly associated with age of first birth for all divisions except for Chattogram and Mymensingh. For example, women residing in Rangpur, Khulna, Rajshahi, and Barisal divisions were 80, 43, 42, and 21% more likely to be married as a child (<18) compared to women who resided in the Dhaka division. However, women who lived in the Sylhet division were 43% significantly less likely to have adolescent motherhood (AOR = 0.57, 95% CI = [0.46, 0.69]) compared to women who resided in the Dhaka division. Women owning a mobile phone were 14% less likely to be married during their childhood (<18) compared to the women who did not own a mobile phone (AOR = 0.86, 95% CI = [0.76, 0.97]).

Both post-marriage study continuation and opinion on preferred time of marriage were found to be significantly associated with age at first marriage and age at first birth (*p* < 0.005) after controlling for relevant sociodemographic factors (Table 5). Compared to women married when adult (≥18 years), those who were married as a girl-child (<18 years) were 36% significantly less likely to continue studies after marriage (AOR = 0.64, 95% CI = [0.50, 0.81]). Similarly, adolescent motherhood (<18 years) led to 55% higher odds of post-marriage discontinuation of education compared to those who became mothers at ≥18 years of age (AOR = 0.45, 95% CI = [0.37, 0.55]). Those who married before 18 years of age or became a mother before 18 years of age were 9.41 and 2.20 times significantly more likely to prefer a later marriage compared to their adult counterparts (≥18 years), respectively (Table 5).

## 4. Discussion

The purpose of this study was to assess the risk factors associated with child marriage and early childbearing among women aged 15–49 years living in Bangladesh, and to evaluate the consequence of child marriage and early childbearing using latest DHS-VII data. Also, the final goal of this study was to identify the vulnerable group of women in Bangladesh through the evaluation of significant risk factors which could prompt actionable changes including interventions from the policy makers’ point of view. This study contributes to the existing literature on child marriage and age at first birth and their consequences in several ways including investigation of whether child marriage and childbearing age were associated with discontinuation of education and women’s opinion on preferred time of marriage in Bangladesh, which to our knowledge has not yet been empirically explored.

This study found that, on average, girls in Bangladesh were at high risk of being married as children and being a mother of a child during their childhood as both rates were significantly high compared to most of the LMICs in the region. Average age of first marriage and average age of first birth were found to be much lower and have not been significantly improved during the last decade [28,36]. The current study also revealed that the distribution of age of first marriage and age of first birth were similar for the younger (≤25 years) and older (>25 years) groups of the surveyed women, which portrays a worrying picture of almost no improvement over two generations.

Conforming to the literature, child marriage, and early child bearing were found to be significantly associated with a number of socio-economic factors, including education, religion, area of residence (urban/rural), partner’s education, and divisions [8,23,26,27,37,38,39,40,41,42,43]. Further analysis revealed that post-marriage study continuation and opinion on preferred time of marriage for women were significantly associated with age at first marriage and age at first birth where girl-child marriage (<18) and adolescent motherhood (<18) led to increased post-marriage study discontinuation and increased preferred time of marriage. This study found that women who gave birth at <18 years of age were 55% more likely to discontinue education than women who gave birth at 18 years of age or older. These findings on the association between child marriage, early child-bearing, and discontinuation of education attainment are consistent with findings of past studies [24,44].

There are many benefits of girls’ education, including increased self-efficacy and life skills, the opportunity for economic development, and self-empowerment. Low educational attainment is consistently associated with functional limitations [45,46,47]. Early marriage compromises girls’ ability to attend school post-marriage, exposing them to an array of adverse social and health outcomes associated with education cessation [48,49]. Early marriage itself serves as a barrier to post-marriage school attendance due to early childbirth and related child-care responsibilities [44]. It was estimated that the rate of child marriages in Bangladesh could drop by a third when girls are educated and taught job skills [50,51].

Having a child at an early age may arise from and give rise to social consequences that increase the risk of developing chronic diseases, mobility loss in older age, and discontinuation of education [24]. This study finding also demonstrated that women who gave birth at 18 years of age or less had lower educational attainment than women who gave birth at an older age, which is consistent with the findings of the current study [52,53]. With less education, women have fewer employment opportunities and are at greater risk of poverty.

Results of this study indicated that a partner’s educational attainment is an important predictor of age of first marriage and age of first birth for the women in Bangladesh, especially partners with secondary and higher levels of education., which complies with the existing literature [28,54]. With education comes increased awareness on the adverse impacts of early marriage and early childbearing, more importantly educated men are likely to be abide by child marriage law. Awareness from the partner is particularly crucial for the patriarchal society of Bangladesh, as a stronger stance from men could go a long way to curb the rampant girl-child marriage and adolescent motherhood in Bangladesh [23].

Religion was found to be significantly associated with age of first marriage and age of first birth where women from Muslim families were significantly more likely to be married and become a mother at childhood (<18) than women from other religions, which complies with past findings [23,44,55]. The significant differences in the prevalence of age of first marriage and age of first birth among the religious groups reflect differences in traditional beliefs, cultural values and social norms that relate not only to child marriage and early childbearing but also to the broader issue of the perceived value of women and the degree of women’s autonomy [23,44,55]. South and Southeast Asian families, including those from Bangladesh, are typically male dominated and the senior male is generally the house head and thus voice of women is often limited [56]. Often the traditional male-controlled environments are justified by religious values and practices, and consequently drive the everlasting practice of child marriage [57,58]. The influence of religious affiliation as an important independent factor in child marriage practice and early childbearing with Muslim women having a higher rate than women from other religious backgrounds is worth noting, particularly in the context of Bangladesh, given that the majority of the population here are Muslims. Religion plays a crucial role in shaping values and practices at individual, household, and community level, often undermining the existing law, which bars social changes including women’s development [59].

Results of this study revealed that girls from rural areas were at a higher risk of getting early marriage (<18 years) than the girls who resided in urban areas of Bangladesh. The girls in the rural areas of Bangladesh are less invested by the family than boys due to the social contract that girls will be married off early and unlikely to contribute to the economy of the household. Due to the nagging existence of a dowry, which is lower for younger girls during marriage, rural household prefer early marriage of the girl-children [28]. Past studies further demonstrated that poor families consider pursuing higher education for girls as unnecessary and encourage them to marry and take care of families, thereby depriving them of an opportunity to decide their future [49,60]. Appropriate intervention programs need to target long-standing traditional cultural norm baring autonomy of women in the rural areas of Bangladesh.

Consistent with the existing literature, this study found that geographical location had significant effects on girl-child marriage and adolescent motherhood in Bangladesh [23,59]. Sylhet division had the lowest percentage of age at first marriage at age <18 years and age at first birth at age <18 years among the eight divisions where these rates were highest in Rajshahi and Ranpur. Some studies have found that heterogeneity in economic growth between Eastern and Western parts of Bangladesh as well as cultural differences [61]. The impact of outmigration in Sylhet and increasing structural growth could play a role here. While a few educated guesses, such as the cultural difference between northern and southern parts of Bangladesh, could be made for the geographic heterogeneity; the survey did not provide any in-depth information for reasonable explanations.

Wealth index was not found to be significantly associated with child marriage and adolescent motherhood, which conformed with previous national survey findings [28]. Contrary to our hypothesis, exposure to media was not found to be a significant predictor of child marriage and adolescent motherhood among women in Bangladesh. The insignificant impact of the wealth index could be due to the economic development that occurred during the last decade and increased employment opportunities created for girls through the garments industry in Bangladesh [62,63]. However, mobile ownership was a predictor of age at first birth, which provides a possible avenue for information dispersion to vulnerable groups of women and future scopes for interventions.

There were several strengths to this study. This study was one of the few that has so far examined some determinants of girl-child marriage and adolescent motherhood among women in Bangladesh using a nationally representative large sample. The study found that child marriage has not decreased in Bangladesh as expected, in fact it has increased in proportion. By evaluating the consequences of child marriage and adolescent motherhood, this study deciphered the deep-rooted cultural obstructions that calls to investing unobserved mediating factors that influence the timing of marriage also influence educational decisions [64,65]. The factors could include parents’ education level and aspirations for children from the in-laws, perceptions on return to schooling, availability of employment opportunities, and the degree of bargaining power of the girl [4].

Few limitations need to be noted. First, although the GIS maps were adjusted, it was limited to specific clusters and could not reflect district-wise information, which would be useful for local policy interventions. Second, data were not available from some remote areas, especially in Chittagong and Sylhet divisions. Third, to complement quantitative data analysis, some qualitative synthesis on socio-economical dynamics and cultural reasoning for prevailing child marriage and adolescent motherhood would help in drawing a causal inference. Fourth, as the data were cross-sectional, the study could only reflect on the association of the factors, but in no way can imply causality. Fifth, the level of education of parents, especially mothers, and the age at which they (mothers) got married could provide a greater insight into cultural impact of child marriage, which could not be included in the current study modelling as the variables were not included in the survey. Furthermore, history of violence in the family could better display the consequence of girl-child marriage, which was also absent in the survey. Six, recall bias and age heaping could compromise some of the scores in marriage age, which could slightly impact the effect sizes of the models. Finally, due to high percentage of missing values, possible consequence variables such as post-marriage working status could not be investigated.

## 5. Conclusions

The study found that the prevalence of girl-child marriage and adolescent motherhood remains persistently high in Bangladesh, if not increased. This study also provides evidence of the association between child marriage, childbearing as a child, and education among women in Bangladesh. In this current study the findings were particularly true for women living in remote areas and were from Muslim religious backgrounds with no education or primary level of education. These findings could improve the targeting of at-risk girls by policy-makers and to help intervene using appropriate intervention programs to keep the country on the track of achieving related SGDs by 2030. Overall, the findings of this study strongly suggest that to implement effective interventions aimed at reducing girl-child marriage and adolescent motherhood for women in Bangladesh, substantial attention must be given to culturally-laden social norms that vary by ethnic groups as well as long-standing religious ideologies.

## Figures and Tables

**Table 1:** Distribution of age of first marriage and age of first birth across the selected sociodemographic factors (*N* = 15,674).

Variables	Age of First Marriage [*N* (%)]		Age of First Birth [*N* (%)]	
	≥18 years	<18 years	≥18 years	<18 years
**Age**				
≤25	1379 (50.3)	6582 (50.9)	2847 (47.6)	5114 (52.7)
>25	1360 (49.7)	6353 (49.1)	3130 (52.4)	4583 (47.3)
*p*-value	0.623		<0.001	
**Education**				
None	217 (7.9)	2384 (18.4)	787 (13.2)	1814 (18.7)
Primary	594 (21.7)	4675 (36.1)	1579 (26.4)	3690 (38.1)
Secondary	916 (33.4)	5035 (38.9)	2154 (36)	3797 (39.2)
Higher secondary or above	1012 (36.9)	841 (6.5)	1457 (24.4)	396 (4.1)
*p*-value	<0.001		<0.001	
**Wealth Index**				
Poorest	302 (11)	2744 (21.2)	867 (14.5)	2179 (22.5)
Poorer	342 (12.5)	2696 (20.8)	958 (16)	2080 (21.4)
Middle	394 (14.4)	2667 (20.6)	1013 (16.9)	2048 (21.1)
Richer	586 (21.4)	2555 (19.8)	1257 (21)	1884 (19.4)
Richest	1115 (40.7)	2273 (17.6)	1882 (31.5)	1506 (15.5)
*p*-value	<0.001		<0.001	
**Area of Residence**				
Rural	1409 (51.4)	8574 (66.3)	3457 (57.8)	6526 (67.3)
Urban	1330 (48.6)	4361 (33.7)	2520 (42.2)	3171 (32.7)
*p*-value	<0.001		<0.001	
**Division**				
Dhaka	494 (18.0)	1816 (14.0)	994 (16.6)	1316 (13.6)
Barishal	240 (8.8)	1432 (11.1)	623 (10.4)	1049 (10.8)
Chattogram	423 (15.4)	1753 (13.6)	842 (14.1)	1334 (13.8)
Khulna	286 (10.4)	1790 (13.8)	751 (12.6)	1325 (13.7)
Mymensingh	244 (8.9)	1469 (11.4)	599 (10.0)	1114 (11.5)
Rajshahi	235 (8.6)	1805 (14.0)	661 (11.1)	1379 (14.2)
Rangpur	246 (9.0)	1755 (13.6)	613 (10.3)	1388 (14.3)
Sylhet	571 (20.8)	1115 (8.6)	894 (15.0)	792 (8.2)
*p*-value	<0.001		<0.001	
**Religion**				
Islam	2206 (80.5)	11,848 (91.6)	5115 (85.6)	8939 (92.2)
Others	533 (19.5)	1087 (8.4)	862 (14.4)	758 (7.8)
*p*-value	<0.001		<0.001	
**Partner’s Age**				
15–34	688 (25.1)	3400 (26.3)	1432 (24.0)	2656 (27.4)
35–50	1483 (54.1)	6054 (46.8)	3029 (50.7)	4508 (46.5)
>50	568 (20.7)	3481 (26.9)	1516 (25.4)	2533 (26.1)
*p*-value	<0.001		<0.001	
**Partner’s Education**				
None	321 (11.7)	3310 (25.6)	1055 (17.7)	2576 (26.6)
Primary	615 (22.5)	4509 (34.9)	1582 (26.5)	3542 (36.5)
Secondary	790 (28.8)	3661 (28.3)	1725 (28.9)	2726 (28.1)
Higher secondary or above	1013 (37.0)	1455 (11.2)	1615 (27.0)	853 (08.8)
*p*-value	<0.001		<0.001	
**Sex of Household Head**				
Male	2435 (88.9)	11,467 (88.7)	5296 (88.6)	8606 (88.7)
Female	304 (11.1)	1468 (11.3)	681 (11.4)	1091 (11.3)
*p*-value	0.732		0.804	
**Working Status**				
No working	1524 (55.6)	5804 (44.9)	3086 (51.6)	4242 (43.7)
Working	1215 (44.4)	7131 (55.1)	2891 (48.4)	5455 (56.3)
*p*-value	<0.001		<0.001	
**Mobile Phone Ownership**				
No	710 (25.9)	5618 (43.4)	2007 (33.6)	4321 (44.6)
Yes	2029 (74.1)	7317 (56.6)	3970 (66.4)	5376 (55.4)
*p*-value	<0.001		<0.001	
**Media Exposure**				
No	711 (26)	4932 (38.1)	1823 (30.5)	3820 (39.4)
Yes	2028 (74)	8003 (61.9)	4154 (69.5)	5877 (60.6)
*p*-value	<0.001		<0.001	
**IPV Considered Justified**				
No	2337 (85.3)	10,142 (78.4)	4945 (82.7)	7534 (77.7)
Yes	402 (14.7)	2793 (21.6)	1032 (17.3)	2163 (22.3)
*p*-value	<0.001		<0.001	
**Involvement in Decision Making**				
No	277 (10.1)	1338 (10.3)	590 (9.9)	1025 (10.6)
Yes	2462 (89.9)	11,597 (89.7)	5387 (90.1)	8672 (89.4)
*p*-value	0.768		0.170	

**Table 2:** Distribution of post-marriage study consequences with age of first marriage and age of first birth (*N* = 5937).

	Post Marriage Study Continuation [*N* (%)]		
	No	Yes	*p*-Value
**Age of First Marriage**			
>18 years	705 (67.8)	335 (32.2)	<0.001
<18 years	4333 (88.5)	564 (11.5)	
**Age of First Birth**			
≥18 years	1710 (74.2)	595 (25.8)	<0.001
<18 years	3328 (91.6)	304 (8.4)	

**Table 3:** Distribution of preferred time of marriage with age of first marriage and age of first birth (*N* = 5937).

	Preferred Time of Marriage Compared to Current Timing [*N* (%)]			
	Earlier	Right Time	Later	*p*-Value
**Age of First Marriage**				
≥18 years	50 (3.8)	950 (91.3)	50 (4.8)	<0.001
<18 years	1081 (22.1)	1510 (30.8)	2306 (47.1)	
**Age of First Birth**				
≥18 years	237 (10.3)	1601 (69.5)	467 (20.3)	<0.001
<18 years	884 (24.3)	859 (23.7)	1889 (52.0)	

**Table 4:** Logistic regression fitted to age of first marriage and age of first birth with sociodemographic factors adjusting for survey weights, and cluster- and strata-wise variations (*N* = 15,674).

	Age of First Marriage		Age of First Birth	
Variables	AOR (95% CI)	*p*-Value	AOR (95% CI)	*p*-Value
**Age (Ref: ≤25)**				
>25			0.66 (0.59, 0.75)	<0.001
**Education (Ref: None or Pre-Primary)**				
Primary	0.84 (0.69, 1.03)	<0.001	1.01 (0.9, 1.13)	0.902
Secondary	0.68 (0.55, 0.84)	<0.001	0.79 (0.69, 0.9)	<0.001
Higher secondary or above	0.13 (0.10, 0.17)	<0.001	0.17 (0.14, 0.2)	<0.001
**Wealth Index (Ref: Poorest)**				
Poorer	0.95 (0.78, 1.16)	0.631	0.94 (0.83, 1.07)	0.368
Middle	1.01 (0.81, 1.26)	0.939	1.05 (0.91, 1.2)	0.506
Richer	0.95 (0.76, 1.18)	0.629	0.97 (0.82, 1.14)	0.692
Richest	0.87 (0.69, 1.11)	0.269	0.89 (0.74, 1.07)	0.202
**Area of Residence (Ref: Rural)**				
Urban	0.82 (0.71, 0.94)	0.005	0.93 (0.84, 1.03)	0.146
**Division (Ref: Dhaka)**				
Barishal	1.57 (1.23, 2.00)	<0.001	1.21 (1.02, 1.43)	0.033
Chattogram	1.07 (0.85, 1.33)	0.575	1.15 (0.98, 1.36)	0.095
Khulna	2.03 (1.62, 2.56)	<0.001	1.43 (1.19, 1.7)	<0.001
Mymensingh	1.47 (1.14, 1.88)	0.003	1.19 (1, 1.43)	0.053
Rajshahi	1.89 (1.53, 2.34)	<0.001	1.42 (1.21, 1.68)	<0.001
Rangpur	2.33 (1.80, 3.01)	<0.001	1.8 (1.5, 2.15)	<0.001
Sylhet	0.40 (0.30, 0.52)	<0.001	0.57 (0.46, 0.69)	<0.001
**Religion (Ref: Islam)**				
Others	0.37 (0.29, 0.46)	<0.001	0.51 (0.42, 0.63)	<0.001
**Partner’s Age (Ref: 15–34)**				
15–34	0.82 (0.72, 0.93)	0.003	0.93 (0.83, 1.04)	0.206
>50	0.95 (0.8, 1.12)	0.536	0.97 (0.82, 1.13)	0.665
**Partner’s Education (Ref: None or Pre-Primary)**				
Primary	0.84 (0.70, 1.00)	0.052	0.95 (0.85, 1.07)	0.422
Secondary	0.76 (0.63, 0.92)	0.005	0.85 (0.75, 0.97)	0.019
Higher secondary	0.52 (0.41, 0.66)	<0.001	0.59 (0.49, 0.72)	<0.001
**Working Status (Ref: No Working)**				
Working	0.96 (0.85, 1.08)	0.511	1.03 (0.95, 1.12)	0.415
**Mobile Phone Ownership (Ref: No)**				
Yes	0.86 (0.76, 0.97)	0.015	0.98 (0.9, 1.07)	0.666
**Media Exposure (Ref: No)**				
Yes	1.00 (0.87, 1.15)	0.997	0.94 (0.85, 1.04)	0.247
**IPV Considered Justified (Ref: No)**				
Yes	1.01 (0.88, 1.17)	0.860	1.06 (0.95, 1.17)	0.295

AOR = Adjusted odds ratio, CI = confidence interval.

**Table 5:** Logistic regression fitted to post-marriage education continuation and preferred time of marriage with age or first marriage and age of first birth (*N* = 5937).

	Post Marriage Study Continuation		Preferred Time of Marriage (Binarized)	
Variables	AOR (95% CI) *	*p*-Value	AOR (95% CI) *	*p*-Value
**Age of First Marriage (Ref: ≥18 Years)**				
<18 years	0.64 (0.50, 0.81)	<0.001	9.41 (6.26, 14.15)	<0.001
**Age of First Birth (Ref: ≥18 Years)**				
<18 years	0.45 (0.37, 0.55)	<0.001	2.20 (1.87, 2.60)	<0.001

AOR = Adjusted odds ratio, CI = confidence interval. * The models were adjusted for respondent’s age, education, wealth index, area of residence, division, religion, partner’s age, partner’s education, working status, mobile phone ownership, and media exposure. Respondents’ education and partners’ education were not included in the models as these variables had less than 15 samples for some categories.

## Data Availability

This article does not contain any studies with human participants performed by any of the authors. Data used in research was attained from National Institute of Population Research and Training (NIPORT), and ICF. 2020, funded by the United States Agency for International Development (USAID). All identification of the respondents was dis-identified before publishing the data. Views expressed in this study do not necessarily reflect those of USAID, the US government, NIPORT or data custodians. The secondary data sets of the current study are available at https://dhsprogram.com/methodology/survey/survey-display-536.cfm. Permission for this project was taken from the Demographic and Health Surveys (DHS) Program authority by the authors.
